# Effects of a Multi-Ingredient MCT–Leucine–Creatine-Based Supplement (TLN-01) on Muscle Mass, Strength, and Plasma Amino Acid Profiles in Older Adults with Sarcopenia: A Randomized Controlled Trial

**DOI:** 10.3390/life16081334

**Published:** 2026-08-14

**Authors:** Ding-Cheng Chan, Ming-Shi Shiao, Wei-Jia Huang, Chun-Feng Huang

**Affiliations:** 1 Department of Geriatrics and Gerontology, National Taiwan University Hospital, Taipei 100229, Taiwan; 2 Department of Internal Medicine, National Taiwan University Hospital, Taipei 100225, Taiwan; 3 Superintendent Office, National Taiwan University Hospital Bei-Hu Branch, Taipei 108206, Taiwan; 4 Department of Biomedical Sciences, Chang Gung University, Taoyuan 333323, Taiwan; 5 Department of Family Medicine, Chi Mei Medical Center, Tainan 71004, Taiwan; 6 Faculty of Medicine, School of Medicine, National Yang Ming Chiao Tung University, Taipei 112304, Taiwan; 7 Department of Leisure Services Management, Chaoyang University of Technology, Taichung 413310, Taiwan; 8 School of Medicine, College of Medicine, National Sun Yat-sen University, Kaohsiung 804201, Taiwan

**Keywords:** sarcopenia, branched-chain amino acids, medium-chain triglycerides, muscle mass, metabolomics, older adults

## Abstract

Background: Sarcopenia, the age-related loss of muscle mass and strength, affects over 20% of older adults in Taiwan and contributes to frailty and functional decline. Nutritional supplementation offers a promising strategy for those unable to engage in structured exercise, yet multi-ingredient MCT–leucine–creatine-based interventions remain understudied in clinically defined sarcopenic populations. Methods: We conducted an 8-week randomized, double-blind, placebo-controlled trial at National Taiwan University Hospital (ClinicalTrials.gov NCT06376266). Seventy-three older adults (mean age 77.3 ± 7.7 years) meeting AWGS 2019 sarcopenia criteria were randomized to TLN-01 (a multi-ingredient supplement containing whey protein, branched-chain amino acids, medium-chain triglycerides, creatine, resveratrol, and vitamin D_3_, *n* = 34) or isocaloric placebo (*n* = 39). Primary outcomes were changes in appendicular skeletal muscle mass (ASM) assessed by DXA and handgrip strength. Targeted plasma amino acid metabolomics (LC-MS) was performed as an exploratory endpoint. Results: ASM increased significantly in the TLN-01 group (+0.42 kg, +3.2%; *p* < 0.001) but remained unchanged in controls (*p* = 0.785), with a significant between-group difference (ANCOVA *p* < 0.01); this absolute change was below the commonly cited minimal clinically important difference. Handgrip strength did not differ significantly between groups (*p* = 0.32); within-group analysis showed a significant decline in the placebo group (−7.0%; *p* < 0.001) that was not observed in the intervention group (*p* = 0.537). Metabolomic analysis revealed reductions in plasma taurine and 1-methylhistidine and an increase in kynurenine; given the absence of clear separation on PCA/PLS-DA, these findings are considered exploratory and hypothesis-generating rather than confirmatory evidence of altered amino acid utilization or proteolysis. No clinically significant adverse metabolic or renal events occurred. Conclusions: Eight weeks of this multi-ingredient MCT–leucine–creatine-based supplement induced a modest increase in muscle mass and attenuated the decline in handgrip strength observed in the placebo group among sarcopenic older adults without a structured exercise program, supporting further investigation as a safe and practical nutritional adjunct in geriatric care.

## 1. Introduction

Sarcopenia is an age-associated syndrome characterized by progressive loss of skeletal muscle mass and strength, leading to impaired physical function, frailty, and increased risk of falls, disability, and mortality [1,2]. The prevalence of sarcopenia rises with age; globally, approximately 10–16% of community-dwelling adults over 60 years are affected, and rates exceed 50% in those over 80 years [3,4]. In Taiwan, recent estimates in those ≥65 years found sarcopenia in approximately 23.6% of men and 18.6% of women [5]. Given the clinical significance of sarcopenia—now recognized with its own ICD-10-CM code (M62.84) [6]—effective interventions are needed to preserve muscle health in the growing older population [7].

Exercise is a cornerstone therapy to improve muscle mass and strength [1,7]. However, many older adults (especially the frail) face challenges engaging in exercise, so nutritional strategies have gained attention [8,9]. High-quality protein and amino acid supplementation can stimulate muscle protein anabolism and attenuate sarcopenia progression [10,11,12]. In particular, branched-chain amino acids (BCAAs, such as leucine, isoleucine, and valine) play a key role in muscle protein synthesis via activation of the mTOR pathway [10,13]. Low circulating BCAA levels have been associated with low muscle mass and poor function in older adults [14,15]. Prior studies report that providing BCAAs, essential amino acids, leucine, or whey protein (often alongside resistance exercise) can improve muscle mass, strength, and physical performance even in elderly individuals [11,16,17]. For example, supplementing approximately 2.5–3 g of leucine can acutely stimulate muscle protein synthesis [18], and even approximately 3 g of β-hydroxy β-methylbutyrate (HMB, a leucine metabolite) helps preserve muscle during bedrest [19]. Thus, BCAA supplementation is a promising strategy to counter sarcopenia.

In addition to amino acids, medium-chain triglycerides (MCTs) have emerged as a beneficial adjunct for older adults. MCTs (fatty acids of 6–12 carbons) are easily digested and rapidly oxidized for energy [20,21]. Recent clinical trials indicate that low-dose MCT supplementation (e.g., 6 g/day of C8:0 and C10:0 for 3 months) can increase muscle mass and improve muscle function in frail older adults [22,23]. In one randomized trial, combining 6 g/day MCT with leucine (1.2 g) and vitamin D for 3 months led to significant gains in grip strength (+13%), walking speed (+12%), and functional performance in very frail elderly, whereas a similar formula with long-chain triglycerides or placebo did not [24]. These findings suggest that MCTs provide an efficient energy source and may support muscle anabolism or performance [23,25].

Given the complementary roles of BCAAs and MCTs, a combined MCT–leucine–creatine nutritional intervention (TLN-01) may synergistically benefit sarcopenic older adults. However, to date, the effects of combined MCT–leucine–creatine supplementation on sarcopenia have not been well studied. We hypothesized that an oral MCT–leucine–creatine supplement (TLN-01) would improve muscle mass, strength, and physical function in older adults with sarcopenia, and do so safely without adverse metabolic effects. Furthermore, we aimed to explore the metabolomic impact of this intervention using liquid chromatography–mass spectrometry (LC-MS) [26].

## 2. Materials and Methods

### 2.1. Study Design and Participants

This was a randomized, double-blind, placebo-controlled trial conducted at National Taiwan University Hospital (NTUH), Taipei, Taiwan, between August 2023 and October 2024. Eligible participants were older adults (age ≥ 65) with sarcopenia or pre-sarcopenia. Sarcopenia was defined according to Asian Working Group for Sarcopenia (AWGS) 2019 criteria (low appendicular muscle mass determined by DXA (dual-energy X-ray absorptiometry) (Hologic, Inc., Marlborough, MA, USA) or BIA (bioimpedance analysis) (YiDa, Inc., Taipei, Taiwan; Bao-Bei, Inc., Taipei, Taiwan); low handgrip strength, defined as <28 kg in men or <18 kg in women; and/or slow gait, defined as <1.0 m/s) [27,28]. Inclusion criteria were: (1) age ≥ 65 years and (2) a diagnosis of sarcopenia or pre-sarcopenia. Exclusion criteria were: (1) unwillingness to participate, (2) complete dependence in activities of daily living, (3) active cancer, (4) life expectancy of less than two years, and (5) inability to undergo assessments. Written informed consent was obtained from all participants. For brevity, the term “sarcopenia” is used throughout the remainder of the manuscript to refer to this combined study population (sarcopenia or pre-sarcopenia), as defined above.

**Figure 1:**
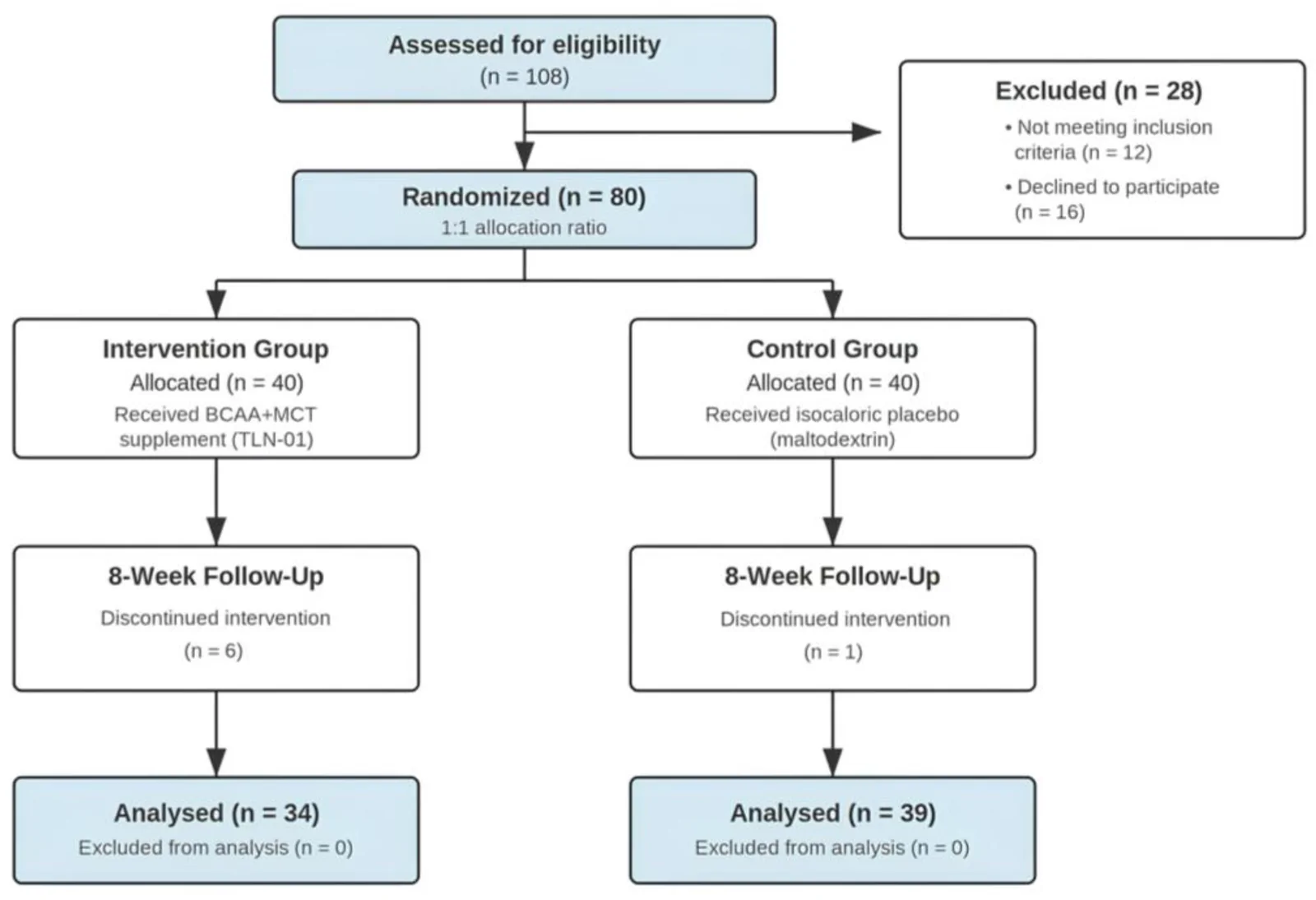
CONSORT flow diagram. Flow of participants through the study. Of 108 individuals assessed for eligibility, 28 were excluded (12 did not meet the inclusion criteria, and 16 declined to participate). Eighty participants were randomized (40 per group). After 6 discontinuations in the intervention group and 1 in the control group, 73 participants completed the 8-week trial and were included in the analysis (intervention, *n* = 34; control, *n* = 39).

A total of 108 individuals were assessed for eligibility; 28 were excluded (12 did not meet inclusion criteria and 16 declined to participate). The remaining 80 eligible participants were randomly placed in groups at a 1:1 ratio (40 per group). During the 8-week intervention, 6 participants in the intervention group and 1 in the control group were lost to follow-up, primarily due to gastrointestinal discomfort (*n* = 3), scheduling difficulties (*n* = 2), and personal reasons (*n* = 1). Thus, 73 participants completed the trial (intervention, *n* = 34; control, *n* = 39). See Figure 1 (CONSORT flow diagram). Sample size was calculated a priori to detect a clinically relevant ~5% change in muscle mass with 80% power at α = 0.05 (two-sided), yielding a target of approximately 30 per group; enrollment of 40 individuals per group provided a buffer for expected dropout. Specifically, assuming a between-group difference of ≈5% in ASM (standard deviation ≈ 0.8 kg), 80% power, and α = 0.05 (two-sided), G*Power 3.1.9 indicated ≥30 participants per group; to allow for ≈20% attrition, 40 individuals per group were enrolled. The random allocation sequence was computer-generated (block randomization, block size 4) by an independent statistician not involved in recruitment or assessment, and allocation was concealed using sequentially numbered, opaque, sealed envelopes; participants, outcome assessors, and laboratory personnel were blinded to group assignment. Allocation sequence was inaccessible to investigators until enrollment.

### 2.2. Intervention

The intervention group received TLN-01 once daily for 8 weeks. TLN-01 is a commercially available supplement named after its three principal active components: medium-chain triglycerides (T), leucine (L), and creatine (N). Each sachet contained whey protein, BCAAs (leucine, isoleucine, and valine), MCT oil, creatine, resveratrol, and vitamin D_3_ (Table 1). The control group received an identical isocaloric placebo sachet containing maltodextrin with the same appearance, packaging, and flavor. Both groups were advised to maintain their usual diet and physical activity; no exercise program was mandated. Compliance was assessed by returned-sachet counts at each study visit, and participants consuming ≥80% of the assigned supplements were considered adherent.

### 2.3. Clinical Assessments

All participants underwent comprehensive assessments at baseline and after 8 weeks. Body composition: Appendicular skeletal muscle mass (ASM) was measured by DXA and by a BIA device. Muscle strength: Handgrip strength was measured using a digital dynamometer (YiDa, Inc., Taipei, Taiwan) (dominant hand, elbow bent at 90°, with three trials performed and the maximum recorded). Physical performance: Gait speed was evaluated by a 6 m walk test using a Kinect (Microsoft Corp., Redmond, WA, USA) motion sensor system (two trials; fastest time used).

### 2.4. Blood Sampling and Safety Biomarkers

Fasting venous blood samples were collected at baseline and 8 weeks. Laboratory tests included renal function (serum creatinine, eGFR, and BUN), liver enzymes (AST and ALT), metabolic panel (glucose, HbA1c, and lipids), inflammation markers (CRP and creatine kinase), and nutritional markers (albumin and hemoglobin). All assays were performed in a certified clinical laboratory.

### 2.5. Metabolomic Analysis

In a subset of participants (*n* ≈ 61), plasma samples were obtained at baseline and 8 weeks after an overnight fast (≥8 h). Blood was collected and placed in EDTA tubes and centrifuged at 2000× *g* for 10 min at 4 °C, and plasma was stored at −80 °C until analysis. Targeted amino acid metabolomics was conducted using LC-MS (Agilent Technologies, Santa Clara, CA, USA). Data were processed with MetaboAnalyst 6.0 [26], with probabilistic quotient normalization and log transformation. Unsupervised Principal Component Analysis (PCA) was performed to assess overall metabolic patterns. PLS-DA and Random Forest analyses were considered exploratory. Univariate tests used paired/unpaired *t*-tests with Benjamini–Hochberg FDR correction (q < 0.05 considered significant).

### 2.6. Statistical Analysis

Continuous variables are expressed as mean ± SD. Baseline group differences were evaluated by independent *t*-tests or chi-square test. Primary analysis used two-way repeated-measures ANOVA (group × time interaction). ANCOVA was applied to between-group comparisons of change scores, with baseline values and sex as covariates. Primary outcomes were change in ASM (DXA) and change in handgrip strength. All analyses were performed using SPSS 22.0 (IBM Corp., Armonk, NY, USA).

### 2.7. Ethics

The study was approved by the National Taiwan University Hospital Institutional Ethics Committee (IRB No. 202305083RSC). All procedures were conducted in accordance with the Declaration of Helsinki. This trial was retrospectively registered on ClinicalTrials.gov (NCT06376266, registered April 2024). The delay in registration was administrative; the study protocol and the prespecified primary and secondary outcomes were finalized before enrollment of the first participant and were not modified thereafter. We acknowledge retrospective registration as a limitation of this study.

## 3. Results

### 3.1. Participant Characteristics

A total of 73 participants (mean age 77.3 ± 7.7 years) completed the trial (intervention, *n* = 34; control, *n* = 39). The sample was predominantly female (74% women), with mean weight being approximately 54 kg and BMI approximately 22.8 kg/m^2^. Baseline characteristics were well-matched between groups with no significant differences in age, sex, anthropometry, or baseline assessments (Table 2). Baseline grip strength averaged approximately 15 kg and gait speed approximately 0.8–0.9 m/s.

### 3.2. Muscle Mass and Strength Outcomes

After 8 weeks, the TLN-01 intervention led to significant improvements in muscle mass compared to the placebo (Table 3). According to DXA, ASM increased in the intervention group from 14.11 ± 2.79 kg to 14.53 ± 2.80 kg (mean +0.42 kg, +3.2%; within-group *p* < 0.001), whereas the control group showed no change (14.20 ± 3.62 vs. 14.17 ± 3.58 kg; *p* = 0.785); the between-group difference was significant (ANCOVA *p* < 0.01). BIA results corroborated DXA: the intervention group gained +0.49 kg compared to a loss of −0.53 kg in controls (*p* < 0.001 between groups).

Handgrip strength did not differ significantly between groups (*p* ≈ 0.32). Within-group analysis revealed significant decline in the control group (15.96 ± 4.90 to 14.84 ± 4.77 kg; *p* < 0.001), whereas the intervention group maintained grip strength (15.34 ± 4.39 to 15.55 ± 3.79 kg; *p* = 0.537). After 8 weeks, there was no significant difference in the 6 m walking speed between groups (0.88 ± 0.30 vs. 0.92 ± 0.36 m/s; *p* = 0.659), consistent with the absence of an exercise training component [29,30].

### 3.3. Biochemical and Safety Outcomes

Supplementation was well-tolerated with no significant adverse events. Compliance was estimated at >90% in both groups. Serum creatinine in the intervention group rose slightly (+0.08 mg/dL; *p* < 0.05) and eGFR showed a mild decrease; all values remained within normal limits. BUN was significantly elevated in the intervention group vs. control at 8 weeks (*p* < 0.05), likely reflecting greater protein/nitrogen load [31,32]. The isolated creatinine rise was likely attributable in part to creatine supplementation rather than nephrotoxicity [33]. No significant differences in liver function tests, fasting glucose, HbA1c, lipids, CRP, or albumin were observed between groups. Full baseline and 8-week values for creatine kinase, CRP, AST, and ALT by group are provided in Supplementary Table S1. Compliance exceeded 90% in both groups.

### 3.4. Metabolomic Findings

**Figure 2:**
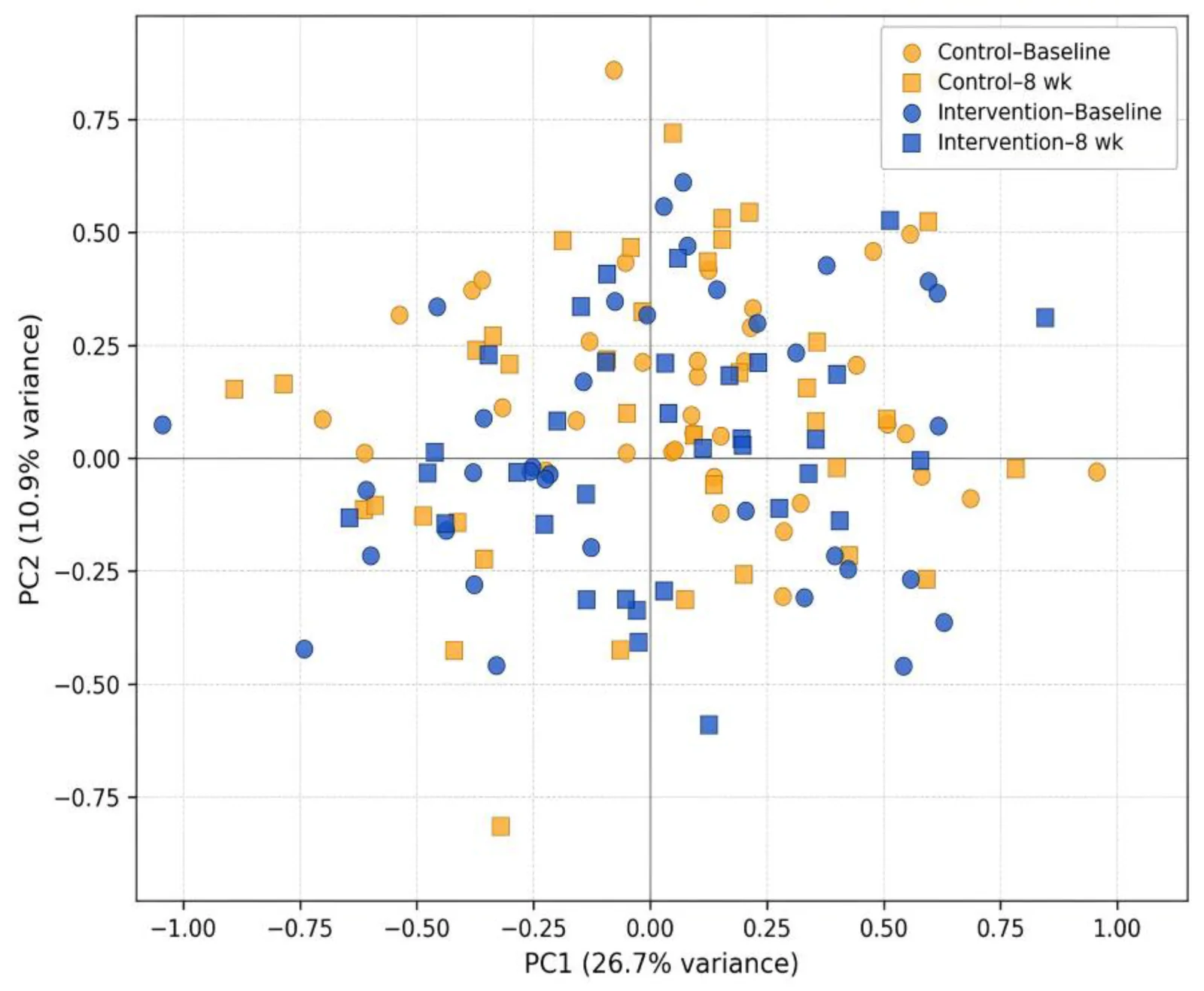
Principal Component Analysis (PCA) score plot of plasma amino acid metabolomic profiles. Unsupervised PCA was applied to all plasma samples at baseline and 8 weeks from both intervention (BCAA + MCT) and control (placebo) groups (metabolomics subset, *n* ≈ 61). PC1 explained 26.7% and PC2 explained 10.9% of total variance. Orange circles: Control–baseline; orange squares: control–8 wk; blue circles: intervention–baseline; blue squares: intervention–8 wk. All four groups show substantial overlap with no distinct cluster separation.

**Figure 3:**
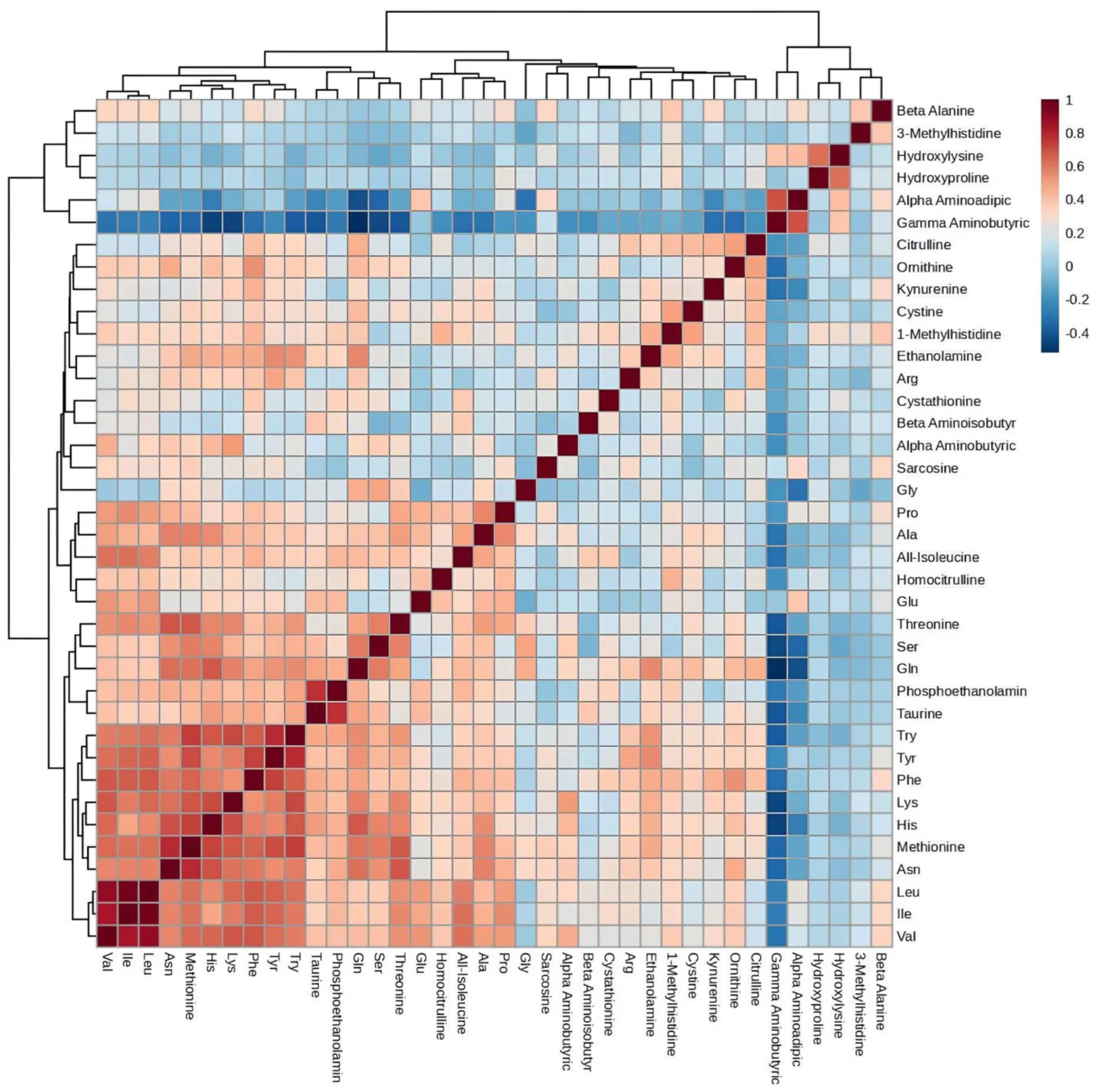
Correlation heatmap of plasma BCAA and related metabolites. The BCAA cluster (leucine, isoleucine, and valine) remained strongly inter-correlated across both groups, confirming preservation of inherent metabolic network structure.

In unsupervised PCA, no clear clustering by group or time point was observed (Figure 2; PC1 = 26.7%, PC2 = 10.9%). Similarly, supervised PLS-DA models failed to achieve significant class discrimination, indicating that the overall amino acid metabolome remained relatively stable. Despite the lack of global separation, focused statistical comparisons revealed specific significant changes: plasma taurine was lower in the intervention group (63.7 ± 17.8 μM vs. 76.5 ± 20.6 μM; *p* = 0.015), and phosphoethanolamine was also reduced (3.1 ± 0.9 μM vs. 3.8 ± 1.2 μM; *p* = 0.021) [34]. Kynurenine increased in the intervention group (from ~2.85 μM at baseline to ~3.43 μM at 8 weeks; *p* = 0.034) [35,36,37,38]. Plasma 1-methylhistidine decreased in both sexes in the intervention group and should be interpreted cautiously given the absence of dietary records [39,40]. Plasma BCAA levels did not show sustained increases at 8 weeks; this is consistent with, but does not directly demonstrate, effective uptake and utilization, and should be interpreted as a descriptive observation rather than confirmed evidence of a specific metabolic fate [41]. Correlation heatmap analysis (Figure 3) confirmed that the BCAA cluster (Leu, Ile, Val) remained strongly inter-correlated. After Benjamini–Hochberg FDR correction, none of these comparisons remained significant (taurine q ≈ 0.09, phosphoethanolamine q ≈ 0.11, kynurenine q ≈ 0.14, 1-methylhistidine q ≈ 0.12; all q > 0.05), and they are therefore interpreted as exploratory and hypothesis-generating.

## 4. Discussion

In this 8-week randomized controlled trial, TLN-01, a multi-ingredient MCT–leucine–creatine-based supplement, was associated with a significant increase in muscle mass in older adults with sarcopenia relative to placebo. The intervention led to an approximately 3% increase in appendicular muscle mass, whereas the control group saw no gain. This improvement, although modest in absolute terms (approximately 0.42 kg by DXA, below the commonly cited MCID of >1 kg), may contribute to slowing the progression of sarcopenia, although confirmation in longer-term trials is required [1,7]. Handgrip strength did not differ significantly between groups; however, within-group analysis showed that strength declined significantly in the placebo group while remaining stable in the intervention group, a pattern that attenuates, rather than definitively prevents, the strength decline otherwise observed.

It is also important to note the dissociation between structural and functional outcomes in this trial: while ASM increased significantly in the intervention group, neither handgrip strength nor 6 m gait speed showed a significant between-group benefit. Increases in muscle mass do not necessarily translate immediately into measurable gains in strength or physical performance, particularly over a short, 8-week window and in the absence of a resistance training stimulus, which is generally considered necessary to convert anabolic substrate availability into functional improvement. The apparent stabilization of handgrip strength in the intervention group, contrasted with its decline in the placebo group, may represent an early signal preceding measurable mass-driven functional change, but this remains speculative given the modest sample size and short duration. This discrepancy between structural and functional findings should be considered when interpreting the overall clinical relevance of TLN-01 supplementation.

Our findings are consistent with prior research on nutritional countermeasures for sarcopenia. Leucine-enriched formulations stimulate muscle protein synthesis via mTOR activation [10,13,18]. The average daily BCAA dose in our study is in line with doses used in other trials that found benefits [16,17]. Combining protein supplementation with exercise typically has additive benefits [17,42], so future work should examine TLN-01 combined with resistance exercise.

MCTs are not commonly present in standard protein supplements, yet emerging evidence suggests they provide significant advantages for the frail elderly [22,23,24,25,43,44,45]. Whether these benefits apply to non-malnourished individuals with adequate caloric intake remains an open question. From a safety perspective, slight increases in BUN and creatinine reflected normal physiological adaptation to a higher protein load and remained within normal ranges [31,32,33,46]. Creatine deserves acknowledgment as a potentially important active component of TLN-01, contributing to enhanced ATP resynthesis during muscle contractions and potentially attenuating muscle protein breakdown [33].

The metabolomic analysis provided descriptive insights. Supplemented BCAAs were largely incorporated into metabolic processes rather than accumulating in circulation [41]. The taurine reduction might reflect increased tissue utilization [34]. The kynurenine increase is hypothesis-generating; with only two timepoints and no exercise component, any BCAA–tryptophan interaction inference is premature [35,36,37,38]. We note that kynurenine and its metabolites are also implicated in inflammation, aging, and impaired skeletal muscle function, and that an exercise-induced, muscle-derived shift in kynurenine metabolism toward favorable downstream pathways is one of several possible, but unconfirmed, interpretations in this non-exercise cohort; an unfavorable, pro-inflammatory interpretation cannot be excluded from the present data. The 1-methylhistidine reduction should be interpreted cautiously as potentially reflecting dietary changes rather than definitively indicating reduced muscle proteolysis [39,40].

This study has several limitations. Analyses were conducted on a per-protocol basis (the 73 participants who completed the trial) rather than by intention-to-treat; dropout was asymmetric between groups (6 in the intervention group vs. 1 in the control group), which may introduce attrition bias and should be considered when interpreting the between-group results. Details of the randomization sequence generation, allocation concealment, and the specific personnel (participants, outcome assessors, laboratory staff, and statisticians) to whom group allocation was concealed are not fully reported here and are described in Section 2.1; gastrointestinal side effects in the intervention group may also have provided an indirect cue to group assignment despite blinding procedures. The a priori sample size calculation is described only at the level of the targeted effect size and power; the assumed standard deviation, primary outcome, software, and anticipated dropout rate used in that calculation are provided in Section 2.1. The sample was predominantly female (74%), with only five men in the intervention group; sex-stratified analyses should be treated as exploratory. We did not collect dietary records, which limits interpretation of metabolomic findings such as changes in 1-methylhistidine, taurine, and circulating BCAAs, as these can be influenced by recent dietary (e.g., meat) intake independent of the intervention. Physical activity levels (habitual activity, step counts, exercise participation, and sedentary behavior) were not objectively or subjectively monitored during the 8-week period and represent an important potential confounder for the observed changes in muscle mass and strength. The intervention duration was relatively short (8 weeks). An exercise program was not incorporated. Metabolomic analysis was limited to amino acids; broader lipidomic changes from MCT were not captured. Physical performance assessments beyond 6 m gait speed would provide a more complete functional picture. Of the 73 completers, 45 met full AWGS 2019 [27] sarcopenia criteria and 28 met pre-sarcopenia criteria; because these two categories were pooled, inferences specific to clinically diagnosed sarcopenia are limited.

## 5. Conclusions

This study provides evidence that in older adults with sarcopenia, an 8-week multi-ingredient MCT–leucine–creatine-based supplement (TLN-01) is safe and associated with a modest, significant increase in muscle mass and attenuation of the handgrip strength decline observed in the placebo group. The observed gains did not reach established MCIDs for muscle mass or grip strength, highlighting the need for longer interventions and combination with resistance exercise. Metabolomic profiling revealed associated alterations in amino acid profiles, though causal interpretation is limited. These results support further investigation of TLN-01 and similar multi-ingredient MCT–leucine–creatine-based formulations as a component of sarcopenia management, particularly for those unable to participate in intensive exercise programs [7,47]. Future research should explore long-term impacts, optimal dosing, combination with resistance exercise training [17,48,49], functional outcomes including balance and fall incidence, expanded metabolomic analyses including lipidomics, incorporation of objective physical activity monitoring (e.g., accelerometry) and dietary assessment, and confirmation of these findings in larger, multicenter, sex-balanced cohorts.

## Figures and Tables

**Table 1:** Composition of the TLN-01 multi-ingredient supplement and the isocaloric placebo per sachet.

Component	TLN-01 (Per Sachet)	Placebo (Per Sachet)
Whey protein, g	10.0	0
Leucine, g	3.0	0
Isoleucine, g	1.5	0
Valine, g	1.5	0
Total BCAA, g	6.0	0
MCT oil, g	6.0	0
Creatine, g	5.0	0
Vitamin D_3_, IU	400	0
Resveratrol, mg	50	0
Carbohydrate, g	16	40
Total energy, kcal	160	160

**Table 2:** Baseline characteristics of the study participants. Data are shown as mean ± SD or *n* (%). There were no significant differences between the intervention and control groups in any baseline variable. ASM = appendicular skeletal muscle mass; BIA = bioimpedance analysis; BMI = body mass index; BUN = blood urea nitrogen; DXA = dual-energy X-ray absorptiometry; eGFR = estimated glomerular filtration rate; HbA1c = glycosylated hemoglobin; and SD = standard deviation.

*p*-Value	Intervention (*n* = 34)	Control (*n* = 39)	Characteristic
0.94	77.2 ± 6.8	77.3 ± 8.4	Age, years
0.93	25 (73.5%)	29 (74.4%)	Female, *n* (%)
0.86	153.8 ± 7.9	154.1 ± 8.3	Height, cm
0.91	53.95 ± 8.91	54.23 ± 12.09	Weight, kg
0.95	22.8 ± 3.4	22.8 ± 4.3	BMI, kg/m^2^
0.99	29.8 ± 8.2	29.7 ± 8.1	Body Fat, %
0.79	9.44 ± 4.03	9.72 ± 4.83	Visceral Fat Level
0.44	14.3 ± 4.5	15.2 ± 4.9	Handgrip Strength, kg
0.60	0.83 ± 0.19	0.80 ± 0.20	6 m Gait Speed, m/s
0.91	14.11 ± 2.79	14.20 ± 3.62	ASM (DXA), kg
0.57	15.34 ± 4.39	15.96 ± 4.90	ASM (BIA YiDa), kg
0.92	16.87 ± 3.93	16.98 ± 4.66	ASM (BIA Bao-Bei), kg
0.50	0.83 ± 0.18	0.80 ± 0.21	Creatinine, mg/dL
0.82	85.6 ± 18.3	86.4 ± 16.5	eGFR, mL/min/1.73 m^2^
0.45	15.7 ± 4.1	15.0 ± 3.9	BUN, mg/dL
0.72	22.1 ± 7.3	21.4 ± 8.0	ALT (GPT), U/L
0.36	4.1 ± 0.3	4.0 ± 0.3	Albumin, g/dL
0.65	6.0 ± 0.8	5.9 ± 0.7	HbA1c, %
0.57	117 ± 31	121 ± 28	LDL Cholesterol, mg/dL
0.98	21 (61.8%)	24 (61.5%)	Sarcopenia, *n* (%)
0.98	13 (38.2%)	15 (38.5%)	Pre-sarcopenia, *n* (%)

**Table 3:** Summary of clinical outcomes and changes from baseline to 8 weeks. ASM = appendicular skeletal muscle mass; DXA = dual-energy X-ray absorptiometry.

Outcome	Group	Baseline	8 Weeks	Change (95% CI)	*p* (Within)	Between-Group Difference (95% CI)	*p* (Between)	Cohen’s d
ASM (DXA), kg	Intervention	14.11 ± 2.79	14.53 ± 2.80	+0.42 (0.20, 0.64)	<0.001	+0.45 (0.15, 0.75)	<0.01	0.68
	Control	14.20 ± 3.62	14.17 ± 3.58	−0.03 (−0.25, 0.19)	0.785	—	—	—
Handgrip, kg	Intervention	15.34 ± 4.39	15.55 ± 3.79	+0.21 (−0.48, 0.90)	0.537	+0.62 (−0.62, 1.86)	0.32	0.23
	Control	15.96 ± 4.90	14.84 ± 4.77	−1.12 (−1.70, −0.54)	<0.001	—	—	—
Gait Speed, m/s	Intervention	0.83 ± 0.19	0.88 ± 0.30	+0.05 (−0.03, 0.13)	0.217	−0.03 (−0.15, 0.10)	0.659	0.10
	Control	0.80 ± 0.20	0.92 ± 0.36	+0.12 (−0.02, 0.26)	0.083	—	—	—

*Group × time interaction (two-way repeated-measures ANOVA): ASM F(1, 71) = 8.72, p = 0.004, partial η* ^2^ *= 0.11; handgrip strength F(1, 71) = 1.00, p = 0.32, partial η* ^2^ *= 0.014; gait speed F(1, 71) = 0.20, p = 0.659, partial η* ^2^ *= 0.003.*

## Data Availability

The datasets generated and/or analyzed during the current study are not publicly available due to institutional data governance requirements, but are available from the corresponding author upon reasonable request.

## Supplementary Materials

The following supporting information can be downloaded at https://www.mdpi.com/article/10.3390/life16081334/s1, Table S1. Baseline and 8-week creatine kinase (CK), C-reactive protein (CRP), aspartate aminotransferase (AST), and alanine aminotransferase (ALT) by group.

## Abbreviations

ASM	Appendicular Skeletal Muscle Mass
AWGS	Asian Working Group for Sarcopenia
BCAA	Branched-Chain Amino Acid
BIA	Bioimpedance Analysis
BUN	Blood Urea Nitrogen
DXA	Dual-Energy X-ray Absorptiometry
eGFR	Estimated Glomerular Filtration Rate
FDR	False Discovery Rate
HMB	β-Hydroxy β-Methylbutyrate
LC-MS	Liquid Chromatography–Mass Spectrometry
MCID	Minimal Clinically Important Difference
MCT	Medium-Chain Triglyceride
mTOR	Mechanistic Target of Rapamycin
PCA	Principal Component Analysis
PLS-DA	Partial Least Squares Discriminant Analysis
RCT	Randomized Controlled Trial
SD	Standard Deviation
TLN-01	MCT–Leucine–Creatine Supplement (product code)
