# Supplementary material for: Effects of a Multi-Ingredient MCT–Leucine–Creatine-Based Supplement (TLN-01) on Muscle Mass, Strength, and Plasma Amino Acid Profiles in Older Adults with Sarcopenia: A Randomized Controlled Trial
Source: Life (Basel). 2026 Aug 14;16(8):1334. doi: 10.3390/life16081334 (PMC13514121; doi:10.3390/life16081334)
Supplement: Supplementary file 1 [file life-16-01334-s001.zip › life-4385218-supplementary.pdf]

Table S1. Baseline and 8-week creatine kinase (CK), C-reactive protein (CRP), aspartate aminotransferase (AST), and alanine aminotransferase (ALT) by group.

Biomarker	Group	Baseline	8 Weeks	*p* (within)	*p* (between)
CK, U/L	Intervention	98 ± 42	112 ± 55	0.21	0.18
	Control	92 ± 38	95 ± 40	0.66	—
CRP, mg/L	Intervention	2.1 ± 1.8	1.9 ± 1.6	0.55	0.30
	Control	2.0 ± 1.7	2.2 ± 1.9	0.48	—
AST, U/L	Intervention	24 ± 7	25 ± 8	0.42	0.51
	Control	23 ± 6	23 ± 7	0.88	—
ALT, U/L	Intervention	22 ± 7	23 ± 8	0.40	0.49
	Control	21 ± 8	21 ± 7	0.85	—
